# A Case of Paradoxical Arthralgia Following Anti-TNF Monoclonal Antibody Administration in a Patient With New-Onset Pediatric Crohn’s Disease

**DOI:** 10.1097/PG9.0000000000000308

**Published:** 2023-04-24

**Authors:** Simone Bellucca, Pier Luigi Calvo, Laura Giugliano, Anna Opramolla

**Affiliations:** From the * Dipartimento di Scienze della Sanità Pubblica e Pediatriche, University of Turin, Italy; † Ospedale Infantile Regina Margherita, Città della salute e della scienza di Torino, Turin, Italy.

**Keywords:** arthralgia, infliximab, azathioprine, adverse event, paradoxical reaction

## Abstract

Anti-TNF antibodies have become a first-line therapy in moderate-to-severe inflammatory bowel diseases. However, there may be some rare paradoxical events and those affecting joints causing severe symptoms need a scrupulous differential diagnosis. When these events occur, it may be necessary to discontinue treatment and shift to another drug class. Herein, we report the case of a 15-year-old boy affected by Crohn’s disease, who developed a paradoxical reaction after the second dose of infliximab. Clinical remission was achieved shifting to budesonide and azathioprine and continuing maintenance therapy with azathioprine alone. To date, no other paradoxical events have occurred.

When anti-Tumor Necrosis Factor (TNF) agents took on a leading role in the management of inflammatory bowel disease, prognosis was significantly improved (1). Although they have a good safety profile, some rare paradoxical adverse events have been reported. These mainly involve the skin (8%–22% of cases) (2,3), but the joints are affected in 1%–2% of cases (4), and differential diagnosis and management of these conditions is particularly challenging.

Herein, we report the case of a paradoxical reaction after infliximab administration in a 15-year-old boy with Crohn’s disease (CD).

## CASE REPORT

A 15-year-old male was admitted to our center with a 2-year history of weight loss, chronic diarrhea, oral aphthae, previous surgically treated perianal fistula, and a significant growth delay (body weight, 34 kg; *z* score, −5.34; height, 159 cm; *z* score, −2.24 on Centers for Disease Control and Prevention growth charts). Laboratory tests revealed severe iron deficiency anemia (Hemoglobin 8.6 g/dL), low folic acid, elevated C-reactive protein (CRP) (53.0 mg/L), and fecal calprotectin (1200 mg/g). He had a blood transfusion at the arrival in the emergency room and iron infusions afterwards during hospitalization. Esophagogastroduodenoscopy was normal, whereas colonoscopy showed severe terminal ileal and ascendant colon involvement (Endoscopic Mayo score 3), confirming CD diagnosis. Given the high Pediatric CD Activity Index (47.5 points: severe), the growth delay, and the history of perianal disease, infliximab therapy was started. A 5 mg/kg dosage was administered, pretreated with chlorphenamine and hydrocortisone, with good tolerance and rapid improvement of abdominal pain and diarrhea. CRP progressively decreased (down to 14.5 mg/L). Two weeks later, the second pretreated infliximab dose was administered (5 mg/kg). Four days after the second infliximab infusion, he presented to the emergency room complaining of severe joint pain and fever. The pain had begun in his wrists bilaterally and later spread to his elbows, hips, and knees. As it was exacerbated by movement, he was barely able to walk. At physical examination, even soft palpation was painful associated with decreased active and passive range of motion of the joints, without erythema or swelling. Ultrasound studies of his knees and hips were negative for synovitis or effusion.

Laboratory tests revealed elevated CRP levels (96.7 mg/L), negative procalcitonin, unmeasurable infliximab trough levels, and positive anti-infliximab antibodies (AIA) (13 ng/mL on ELISA assay). Antinuclear antibodies screening was negative. Prophylactic wide spectrum antibiotic therapy was administered, until such time as cultures were negative. Viral serologies and polymerase chain reactions were also negative. His clinical condition gradually improved, and after 4 days, he was apyretic without any joint pain.

The adverse drug reaction probability scale by Naranjo et al (5) was “probable”. Consequently, suspecting a paradoxical reaction, infliximab was discontinued, and therapy was immediately shifted to azathioprine plus budesonide at 9 mg/day with progressive scaling. Aimed at obtaining a complete clinical remission, azathioprine dosage was progressively increased (up to 2 mg/kg) during the following 2 months, with continuous laboratory controls for tolerance. At the time of writing, 9 months after the onset of symptoms, the boy is in clinical remission, has put on 13 kg (*z* score, –1.02) and grown 4 cm (−0.96), without recurrence of polyarthritis.

## DISCUSSION

As aforementioned, the advent of anti-TNF marked enormous progress in the treatment of inflammatory disorders, such as CD. European Society for Paediatric Gastroenterology Hepatology and Nutrition guidelines indicate anti-TNF factors as first-line therapy in moderate-to-severe disease (6). Anti-TNF therapy has an overall favorable safety profile (7), even if rare paradoxical manifestations have been reported and some of them deserve particular attention. Although most of them involve psoriasiform skin manifestations, a few cases with joint symptoms have been described (8,9). This less frequent adverse event accounts for 1%–2% of CD patients treated with anti-TNF agents. The key pathophysiology proposed in the development of these manifestations is immune dysregulation and type 3 delayed hypersensitivity reaction. This condition is described to be associated with low infliximab trough levels and positive AIA, as in our case. A possible strategy to reduce anti-TNF immunogenicity could be a combo-therapy with AZA, which, unfortunately, was not considered in our patient. High titers of antinuclear antibodies are described in some of these patients, with high levels of anti-dsDNA.

On the contrary, polyarthralgia has multiple etiologies. CD might be associated with polyarthritis and dactylitis, under the spectrum of seronegative spondyloarthropathy, which were excluded on the basis of atypical joint involvement and negative ultrasound. Moreover, since polyarthralgia is one of the late infusion reactions to biologics manifestations, it must also be considered. It occurs >24 hours postinfusion, caused by activation of complement by antigen–antibody immune complexes deposited in blood vessels, skin and joint tissue, and are more common in patients with multiple risk factors for accelerated infliximab drug clearance (eg, a prolonged untreated high inflammatory state and perianal fistulizing disease). It typically manifests with pruritic skin eruptions, fever, malaise, and polyarthralgia; jaw pain is also often reported (10). The differential diagnosis between paradoxical and infusion reaction is particularly complex, given the overlap of their clinical manifestations. In our case, we considered the diagnosis of a paradoxical reaction more likely due to the severity of the arthralgia, the absence of skin lesions and jaw pain, as well as the evidence of low infliximab trough levels with positive AIA.

According to the guidelines, patients with high titers of AIA who do not respond to treatment should be switched to another agent (ie, adalimumab). However, in patients presenting with paradoxical reactions, switching to another anti-TNF agent is not recommended (4). Therefore, our main therapeutic options were new biological agents (ustekinumab or vedolizumab) versus immunomodulator drugs (thiopurines or methotrexate). Since the great clinical experience was gained over years of use of thiopurines (6), a combination of azathioprine and budesonide was chosen. This therapeutic approach was effective in inducing and maintaining clinical and endoscopic remission.

## CONCLUSIONS

Anti-TNF agents represent a cornerstone in CD therapy. Treatment optimization should be implemented (ie, combo-therapy), especially in cases with signs of high drug clearance. Despite a good safety profile, there may be some paradoxical reactions involving joints and, at times, symptoms may be severe. Because of the partial clinical overlap between paradoxical reactions, CD extraintestinal manifestation, and late infusion reaction, differential diagnosis is challenging. The diagnosis should be based on an attentive clinical evaluation and laboratory findings, including drug trough levels and antidrug antibodies. Management is complex and, when there is a suspicion of paradoxical reaction, the treatment is to be switched to another pharmacological class therapy.

## ACKNOWLEDGMENT

Patient’s parents gave their informed consent to authors for this submission.
